# Sarcopenia as potential biological substrate of long COVID‐19 syndrome: prevalence, clinical features, and risk factors

**DOI:** 10.1002/jcsm.12931

**Published:** 2022-06-14

**Authors:** Anna Maria Martone, Matteo Tosato, Francesca Ciciarello, Vincenzo Galluzzo, Maria Beatrice Zazzara, Cristina Pais, Giulia Savera, Riccardo Calvani, Emanuele Marzetti, Maria Camprubi Robles, Maria Ramirez, Francesco Landi, Gemelli Against COVID‐19 Post‐Acute Care Team

**Affiliations:** ^1^ Fondazione Policlinico Universitario Agostino Gemelli IRCCS Rome Italy; ^2^ Research and Development Abbott Nutrition Granada Spain

**Keywords:** Sarcopenia, Long COVID‐19, Personalized medicine, Frailty, Elderly

## Abstract

**Background:**

Severe clinical pictures and sequelae of COVID‐19 disease are immune mediated and characterized by a ‘cytokine storm’. Skeletal muscle has emerged as a potent regulator of immune system function. The aim of the present study is to define the prevalence of sarcopenia among COVID‐19 survivors and the negative impact of sarcopenia on the post‐acute COVID‐19 syndrome and its related risk factors.

**Methods:**

A total of 541 subjects recovered from COVID‐19 disease were enrolled in the Gemelli Against COVID‐19 Post‐Acute Care between April 2020 and February 2021. They underwent a multidisciplinary clinical evaluation and muscle strength and physical performance assessment.

**Results:**

Mean age was 53.1 years (SD 15.2, range from 18 to 86 years), and 274 (51%) were women. The prevalence of sarcopenia was 19.5%, and it was higher in patients with a longer hospital stay and lower in patients who were more physically active and had higher levels of serum albumin. Patients with sarcopenia had a higher number of persistent symptoms than non‐sarcopenic patients (3.8 ± 2.9 vs. 3.2 ± 2.8, respectively; *P* = 0.06), in particular fatigue, dyspnoea, and joint pain.

**Conclusions:**

Sarcopenia identified according to the EWGSOP2 criteria is high in patients recovered from COVID‐19 acute illness, particularly in those who had experienced the worst clinical picture reporting the persistence of fatigue and dyspnoea. Our data suggest that sarcopenia, through the persistence of inflammation, could be the biological substrate of long COVID‐19 syndrome. Physical activity, especially if associated with adequate nutrition, seems to be an important protective factor.

## Introduction

One year after the appearance on the world scene of the SARS‐CoV‐2 infection, there are increasing evidence that it has a systemic, inflammatory pathogenesis.^1^ The biological and clinical course of COVID‐19 is characterized by three phases, showing at the onset an early tropism for the upper respiratory system, which results at most in few symptoms, such as mild fever and fatigue. Thereafter, it replicates in the lower respiratory tract, and the patient complains of cough and dyspnoea. Finally, around the 10th–14th day from the onset of symptoms, it causes viraemia with subsequent attack against all organs that express angiotensin‐converting enzyme‐2 receptors such as the heart, kidney, gastrointestinal tract, and blood vessels with variable clinical manifestations in terms of site and severity.^2^, ^3^

The distinctive feature of those subjects who develop severe disease manifestations is not the extent of the viral damage, but the immune injury mediated by an exaggerated inflammation supported by the so‐called ‘cytokine storm’.^4^ The progression of COVID‐19 is associated with a continuous decrease in lymphocyte count and significant elevation of neutrophils and inflammatory markers including C‐reactive protein, IP‐10, MCP1, MIP1A, TNF‐α, interleukin‐6, and ferritin.^2^, ^5^, ^6^ Cytokines and chemokines attract many inflammatory cells, such as neutrophils and monocytes, resulting in excessive infiltration of them into tissues. This dysregulated and/or exaggerated cytokine and chemokine response by infected cells plays a key role in the pathogenesis of SARS‐CoV‐2, and it is responsible for the massive prevalence of catabolic pathways that are observed during the acute phase of illness and its sequelae. In fact, at biochemical level, many authors have reported up‐regulation of apoptosis, autophagy, and p53 pathways in peripheral blood mononuclear cells of COVID‐19 patients.^7^

Skeletal muscle is the most important ‘metabolic controller’ of our body. It is well known that muscle is the main site of glucose and fatty acid metabolism, through peroxisome proliferator‐activated receptors, and thermoregulation. Furthermore, it is important to highlight that muscle has emerged as a potent regulator of immune system function.^8^ Sarcopenia is a progressive and generalized skeletal muscle disorder (which include altered muscle strength and function) that is associated with increased likelihood of adverse outcomes.^9^, ^10^ When it occurs, in addition to complications as falls and disability, increased infections and significant alterations in the immune system are observed. While increased evidence had shown that lower muscle mass is independently associated with intensive care unit admission and hospital mortality,^11^, ^12^ there are no studies on the impact of COVID‐19 on muscle and on the incidence of sarcopenia in post‐COVID‐19.

The aim of the present study is to provide a better insight into the comprehension of the prevalence of sarcopenia (according to the new EGSWOP2 definition) among COVID‐19 survivors and the negative impact of sarcopenia on the long COVID‐19 syndrome and its related risk factors.

## Materials and methods

The Gemelli Against COVID‐19 Post‐Acute Care (GAC19‐PAC) project was an initiative developed by the Department of Geriatrics, Neuroscience and Orthopedics of the Catholic University of the Sacred Heart (Rome, Italy) to better understand what happens in survival COVID‐19 patients and how the virus impacted their health and quality of life. Beginning on 21 April 2020, the Fondazione Policlinico Universitario Agostino Gemelli IRCCS (Rome, Italy) established an outpatient service for individuals who suffered the SARS‐CoV‐2 infection. This outpatient service—called ‘Day Hospital Post‐COVID‐19’—is currently ongoing with the aims to expand the knowledge of COVID‐19 and its impact on health status and care needs as well as to promote healthcare strategies to treat and prevent the clinical consequence of SARS‐CoV‐2 infection across different organs and systems. Further details about the post‐acute outpatient service and evaluation of the patients have been described elsewhere.^13^, ^14^, ^15^

### Study sample

Between 21 April 2020 and 28 February 2021, 623 individuals officially recovered from COVID‐19 were followed in Day Hospital Post‐COVID‐19. For the present study, 82 subjects were excluded for missing values in the variables of interest; as a consequence, a sample of 541 subjects was considered.

At the follow‐up visit, all these patients met the World Health Organization (WHO) criteria for discontinuation of quarantine: no fever for three consecutive days, improvement in other COVID‐19‐related symptoms, and two negative tests for the SARS‐CoV‐2 virus 24 h apart.

### Data collection

Patients were offered a comprehensive medical assessment with detailed COVID‐19‐related history and physical examination. A multidisciplinary approach, including internal medicine, geriatric, ophthalmological, otolaryngologic, pneumological, cardiological, neurological, immunological, and rheumatological evaluations, was put in place for a comprehensive assessment of all the possible damages caused by the SARS‐CoV‐2 virus.^16^ All clinical parameters, including clinical and pharmacological history, lifestyle including smoking status and physical activity, and anthropometric measures, were collected in a structured electronic data collection system. Smoking habit was categorized as current or never/former smoker. Body weight was measured through an analogue medical scale. Body height was measured using a standard stadiometer. Body mass index was defined as weight (kg) divided by the square of height (m). Regular participation in physical activity was considered as involvement in exercise training at least twice a week.

The specific symptoms potentially correlated to COVID‐19 were obtained using a standardized questionnaire in which the patient was asked about the presence or absence of the symptom and more than one symptom could be reported.^17^ Patients were asked to recount symptoms retrospectively during the clinic visit and to confirm the persistence of them. A specific focus has been paid to collect information and data about signs and symptoms COVID‐19 related: cough, fatigue, diarrhoea, headache, smell disorders, dysgeusia, red eyes, joint pain, short of breath, loss of appetite, sore throat, and rhinitis.

According to the WHO classification,^18^ the COVID‐19 severity has been defined as (i) patient at home and no hospitalization, (ii) patient hospitalized without oxygen support, (iii) patient hospitalized with oxygen support by Venturi mask, (iv) patient hospitalized with oxygen support by non‐invasive ventilation or continuous positive airway pressure, and (v) patient hospitalized in intensive care unit with invasive ventilation.

### Muscle strength and physical performance assessment

Muscle strength was assessed by handgrip strength, which was measured by using a dynamometer (North Coast Hydraulic Hand Dynamometer, North Coast Medical, Inc, Morgan Hill, CA). Participants performed one familiarization trial and one measurement trial with each hand, and the result from the stronger side was used for the analyses.^11^

Participants' physical performances and oxygen saturation were evaluated by the chair stand test and the 6 min walking test. Subjects were asked to stand up from a chair with their arms folded across the chest for one minute as quickly as possible. A standard armless chair was used, usually 43–47 cm in height. The back of the chair was stabilized against a wall to ensure safety and stability. The number of times the patient completed the stand and sit cycle was recorded; higher number reflected better performance.^19^ The 6 min walking test was performed along a distance of 20 m, and the distance covered in the given time was recorded in metres; greater number of metres reflected better performance.

### Sarcopenia definition

According to the most recent EWGSOP2 consensus definition,^9^ low muscle strength is considered as the primary parameter of sarcopenia. Sarcopenia is probable when low muscle strength is detected. The EWGSOP2 sarcopenia cut‐off points for low strength by grip strength were considered. Hence, subjects over 65 years of age were defined to be affected by probable sarcopenia when handgrip strength was <27 kg in male and <16 kg in female, respectively.^9^ For the subjects in the lower age groups, the cut‐off values for sex and age previously identified in a large sample of non‐hospitalized subjects living in the community (Lookup 7+ sample) were used.^12^

### Ethical approval and manuscript preparation

This study has been approved by the Catholic University/Fondazione Policlinico Gemelli IRCCS Institutional Ethics Committee (protocol ID number: 0013008/20).^17^ Written informed consent has been obtained from the participants. The manuscript was prepared in compliance with the STrengthening the Reporting of OBservational studies in Epidemiology (STROBE) reporting guidelines for observational studies.

### Statistical analyses

Continuous variables were expressed as mean ± standard deviation (SD) and categorical variables as frequencies by absolute value and percentage (%) of the total. Descriptive statistics were used to describe clinical characteristics of the study population according to sarcopenia status. The differences in proportions and the means of covariates between subjects with and without sarcopenia were assessed using Fisher's exact test and *t*‐test statistics, respectively.

Cox proportional hazard models with robust variance estimates were used to assess the association between clinical and functional characteristics and sarcopenia prevalence. Candidate variables to be included in the Cox model were selected on the basis of biological and clinical plausibility as risk factor for sarcopenia. To identify factors independently associated with prevalent sarcopenia, we first estimated crude odds ratio (OR) and its 95% confidence interval (CI). A multivariable Cox model was computed including all the variables that were associated with the outcome at *α* level of 0.05, after adjustment for age and gender. Model 1 included all the variables of interest and the WHO severity score of COVID‐19 by comparing the risk of hospitalization with staying at home. Model 2 included the same variables as Model 1 by removing COVID‐19 severity score and including length of hospital stay. Consequently, all subjects were included in Model 1 (*n* = 541), while in Model 2, only hospitalized subjects were considered (*n* = 332).

All analyses were performed using SPSS software (Version 11.0, SPSS Inc., Chicago, IL).

## Results

**Figure 1:**
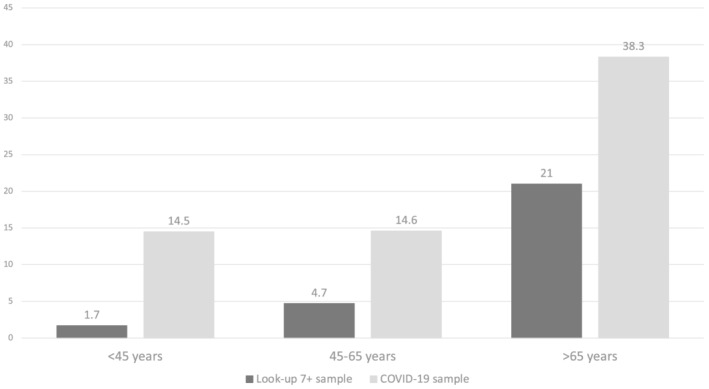
Prevalence of sarcopenia according to age group and study sample (COVID‐19 sample vs. Lookup 7+ community sample).

Mean age of 541 subjects included in the present study was 53.1 years (SD 15.2, range from 18 to 86 years), and 274 (51%) were women. The prevalence of sarcopenia was 19.5%, with a significant difference between men and women (15.7% vs. 23.3%, respectively; *P* = 0.01). The average days from onset of COVID‐19 to follow‐up visit were substantially similar among subjects with sarcopenia vs. non‐sarcopenic subjects (87.5 ± 47.4 vs. 95.3 ± 51.7, respectively; *P* = 0.1). In COVID‐19 survivors under 45 years of age, sarcopenia was present in 14.5%; in subjects between 45 and 65 years, it was 14.6%; while in subjects over 65, it was 38.3%. *Figure* 1 shows the prevalence of sarcopenia according to different age groups by comparing the rates observed in COVID‐19 survivors with those of the Lookup 7+ project comprising more than 11 000 non‐hospitalized subjects living in community. The sarcopenia rate in the subjects affected by COVID‐19 was always significantly higher than in the subjects enrolled in the Lookup 7+ project.

Characteristics of the study population according to the sarcopenia status are summarized in *Table* 1. Compared with participants without sarcopenia, those diagnosed with sarcopenia were significantly older and had greater prevalence of hypertension, diabetes, and chronic obstructive pulmonary disease. Subjects without sarcopenia at the time of follow‐up visit were more physically active and had higher levels of serum albumin and haemoglobin. Finally, the prevalence of sarcopenia was significantly higher in hospitalized subjects, particularly among those subjects who needed oxygen therapy (i.e. non‐invasive ventilation and continuous positive airway pressure) and invasive ventilation. Among subjects who had been hospitalized, the length of hospital stay was significantly longer among subjects with sarcopenia at follow‐up time than among those without sarcopenia.

**Table 1:** Characteristics of study population according to sarcopenia

Characteristics	Total sample (*n* = 541)	No sarcopenia (*n* = 435)	Sarcopenia (*n* = 106)	*P*
General and clinical characteristics				
Age (years)	53.1 ± 15.2	51.5 ± 14.6	59.9 ± 15.9	<0.001
Gender				
Male	267 (49)	225 (84)	42 (16)	0.01
Female	274 (51)	210 (77)	64 (23)	
Education (years)	14.5 ± 4.1	14.7 ± 3.9	13.7 ± 5.0	0.04
Smoking habit	43 (9)	33 (7)	10 (9)	0.34
Physically active	308 (58)	258 (61)	50 (47)	<0.01
Hypertension	158 (29)	115 (26)	43 (41)	<0.01
Heart failure	11 (2)	9 (2)	2 (2)	0.63
Diabetes	42 (8)	25 (6)	17 (16)	0.001
Renal failure	15 (3)	10 (2)	5 (5)	0.15
COPD	43 (8)	26 (6)	17 (16)	0.001
Cancer	13 (2)	10 (2)	3 (3)	0.48
BMI (kg/m^2^)	25.7 ± 4.5	25.6 ± 4.5	25.8 ± 4.2	0.75
Severity of COVID‐19 during acute phase				
Home	209 (39)	178 (85)	31 (15)	<0.001
Hospital—no O_2_ support	103 (19)	91 (88)	12 (12)	
Hospital—O_2_ support	149 (27)	117 (78)	32 (22)	
Hospital—NIV or CPAP	53 (10)	37 (70)	16 (30)	
Hospital—invasive ventilation	27 (5)	12 (44)	15 (56)	
Length of hospital stay (days)	16.3 ± 13.6	13.6 ± 10.3	26.5 ± 19.0	<0.001
Haematological parameters				
Albumin	42.8 ± 3.6	43.1 ± 3.0	41.3 ± 3.6	<0.001
Haemoglobin (g/dL)	13.8 ± 1.4	13.9 ± 1.3	13.5 ± 1.5	0.02
Vitamin D (μg/L)	26.0 ± 12.6	26.5 ± 13.0	24.0 ± 10.7	0.06
C‐reactive protein (mg/L)	2.6 ± 6.0	2.4 ± 5.8	3.3 ± 6.7	0.15
Physical function measurements				
6 min walking test (m)	536.7 ± 95.7	546.3 ± 87.7	494.0 ± 107.0	<0.001
Chair stand test (number of repetitions)	26.2 ± 8.9	26.6 ± 8.4	24.4 ± 10.7	0.05

BMI, body mass index; COPD, chronic obstructive pulmonary disease; CPAP, continuous positive airway pressure; NIV, non‐invasive ventilation; SD, standard deviation.

Data are given as number (%) for gender, smoking, physical activity, diseases, place, oxygen support, and invasive ventilation; for all the other variables, means ± SD are reported. Physically active: physical exercise at least twice a week.

The presence of sarcopenia was associated with reduced physical performance. In particular, subjects with sarcopenia walked about 60 m less during the 6 min walking test than non‐sarcopenic ones (494 vs. 546 m, respectively; *P* < 0.001). Similarly, sarcopenic subjects had reduced number of repetitions during the chair stand test performance (24 vs. 26, respectively; *P* = 0.05).

**Figure 2:**
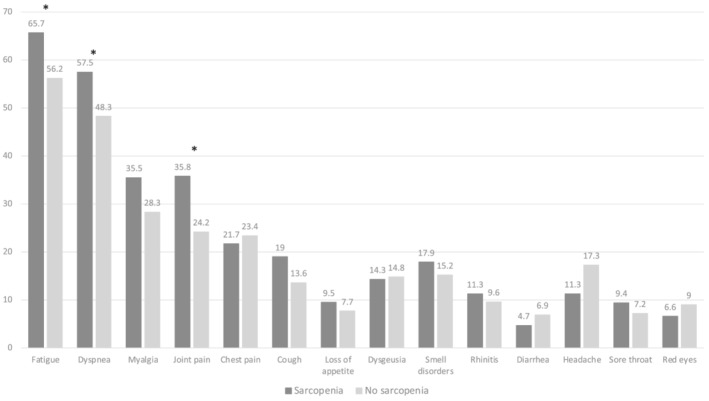
Prevalence of persistent COVID‐19‐related symptoms according to the presence of sarcopenia (^*^≤0.05).

*Figure* 2 shows the prevalence of persistent COVID‐19‐related symptoms according to the presence of sarcopenia. Overall, patients with sarcopenia had on average a higher number of persistent symptoms than non‐sarcopenic patients (3.8 ± 2.9 vs. 3.2 ± 2.8, respectively; *P* = 0.06). In particular, a significantly higher percentage of fatigue (65% vs. 56%; *P* = 0.04), dyspnoea (57% vs. 48%; *P* = 0.05), and joint pain (36% vs. 24%; *P* = 0.01) was observed among sarcopenic subjects than in non‐sarcopenic subjects. Even though not statistically significant, loss of appetite during the acute phase of COVID‐19 is higher among sarcopenic subjects compared with subjects without sarcopenia.

Finally, Cox proportional hazard models were used to assess the association between clinical and functional characteristics and sarcopenia prevalence. After multivariable adjustment (Model 1), as expected, the likelihood of being sarcopenic increased progressively and independently with advancing age [prevalence ratio (OR) 1.02; 95% CI 1.01–1.04], and the risk was significantly higher among female participants (OR 1.88; 95% CI 1.06–3.33). Sarcopenia was associated with diabetes (OR 2.34; 95% CI 1.10–4.96) and with the severity of COVID‐19 as expressed by the need of invasive ventilation (OR 2.78; 95% CI 1.04–7.43). Conversely, a decreased probability of being sarcopenic at the follow‐up visit was detected among subjects with higher levels of serum albumin (OR 0.90; 95% CI 0.83–0.98) and involved in regular physical activity (OR 0.64; 95% CI 0.39–0.99). Furthermore, when multivariate analysis was restricted to hospitalized patients, a longer length of hospital stay was significantly associated with an increased risk of developing sarcopenia (OR 1.05; 95% CI 1.02–1.07) (*Table* 2).

**Table 2:** Unadjusted and adjusted association (OR and 95% CI) between potential risk factors and the presence of sarcopenia

Characteristics	Unadjusted	Model 1	Model 2
	OR (95% CI)	OR (95% CI)	OR (95% CI)
Age (years)	1.04 (1.02–1.05)	1.02 (1.01–1.04)	1.04 (1.01–1.07)
Gender (female)	1.63 (1.06–2.51)	1.88 (1.06–3.33)	1.77 (0.85–3.69)
Education	0.94 (0.90–0.99)	0.98 (0.92–1.04)	0.92 (0.85–1.00)
Physically active	0.57 (0.37–0.88)	0.64 (0.39–0.99)	0.75 (0.40–1.41)
COPD	3.00 (1.56–5.77)	2.13 (0.99–4.96)	2.24 (0.89–5.63)
Diabetes	3.13 (1.62–6.04)	2.34 (1.10–4.96)	2.66 (1.06–6.64)
Hypertension	1.89 (1.22–2.95)	0.66 (0.36–1.22)	0.65 (0.31–1.34)
Albumin	0.84 (0.79–0.90)	0.90 (0.83–0.98)	0.92 (0.82–1.03)
Haemoglobin	0.84 (0.72–0.97)	0.87 (0.71–1.07)	0.91 (0.69–1.19)
Severity of COVID‐19			
Home	1.0 (referent)	1.0 (referent)	—
Hospital—no O_2_ support	0.75 (0.37–1.54)	0.64 (0.30–1.39)	
Hospital—O_2_ support	1.57 (0.90–2.71)	0.73 (0.37–1.43)	
Hospital—NIV or CPAP	2.48 (1.23–4.99)	0.99 (0.43–1.04)	
Hospital—invasive ventilation	7.17 (3.06–16.78)	2.78 (1.04–7.43)	
Length of hospital stay (days)	1.06 (1.04–1.08)	—	1.05 (1.02–1.07)

CI, confidence interval; COPD, chronic obstructive pulmonary disease; CPAP, continuous positive airway pressure; NIV, non‐invasive ventilation; OR, odds ratio.

## Discussion

In the present study, we explored the prevalence of sarcopenia, defined using the new EGSWOP2 operational definition,^9^ among a large sample of COVID‐19 survivors. The prevalence of sarcopenia was 19.5%, a particularly relevant data compared with the one in the general population. After 3 months on average from the onset of COVID‐19, a large number of patients still have sarcopenia and not fully recovered, compared with those living in community who did not have COVID‐19. In fact, in the largest and most recent Italian database of muscle values collected from an unselected sample of subjects living in community, the prevalence of sarcopenia was 8.6%.^11^ It is important to highlight that this study involved more than 11 000 subjects and the mean age of participants was 55.6 years, very similar to the present sample of COVID‐19 survivors.^11^ The differences in terms of sarcopenia prevalence between general population and patients recovered by COVID‐19 are macroscopic at all ages. However, the high prevalence among older patients may become an emergency issue if we consider that sarcopenia is considered the biological substrate of physical frailty and the pathway whereby the consequences of physical frailty develop. In fact, physical frailty is associated with negative outcomes such as falls, mobility disability, loss of independence, and death.^20^, ^21^ In this respect, it is important to underline that, compared with participants without sarcopenia, those diagnosed with sarcopenia had greater prevalence of hypertension, diabetes, and chronic obstructive pulmonary disease; all these diseases, like sarcopenia, have an inflammatory pathogenesis.

We explore the association of sarcopenia with the long COVID‐19‐related symptoms, too. A significantly higher percentage of fatigue and dyspnoea was observed in sarcopenic subjects than in non‐sarcopenic subjects. This is one of the most significant results of present research considering that, as recently reported in our previous study,^17^ among patients who had recovered from COVID‐19, more than 85% reported persistence of at least one symptom, particularly fatigue, dyspnoea, and joint pain. These symptoms alone or variously combined with others, such as ‘brain fog’, sleep disturbances, attention deficit, and generalized and discontinuous muscle pain, configure the so‐called ‘long post‐COVID‐19 syndrome’,^17^ which is emerging more and more as one of the most important challenges of healthcare systems.^22^ This cluster of clinical features closely resembles the typical features of the fibromyalgia and chronic fatigue syndrome and, most surprisingly, is not associated with psychiatric disorders or residual functional and/or structural deficits in pulmonary parenchyma.^23^ These two conditions share a common pathophysiological aetiology identified as central sensitization,^24^ whose pathogenesis is precisely inflammation mediated. Central sensitization occurs when the peripheral response of the spinal neuron becomes independent of the injurious insult received. The state of sensitization is maintained and enhanced by the release of pro‐inflammatory cytokines by glial cells, which, when hyperactivated, cause a real neuroinflammation.^25^, ^26^

**Figure 3:**
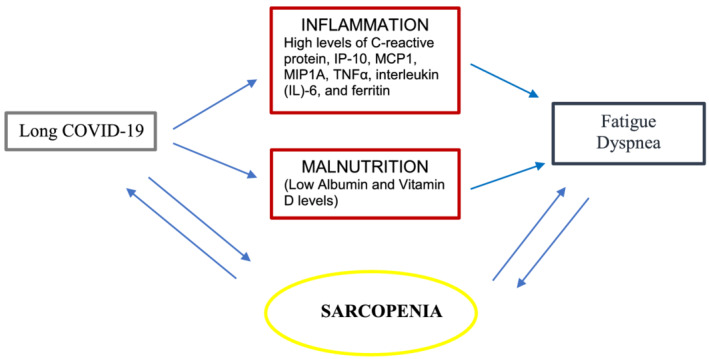
Interaction between COVID‐19 and sarcopenia.

Given these considerations, we can hypothesize that sarcopenia—which in turn is mediated by the persistence of interleukins systemic inflammation—may be the biological substrate of fatigue, the main symptom of the long COVID‐19 syndrome, as well as of dyspnoea, which can be considered a fatigue affecting the respiratory muscles (*Figure* 3).

Finally, we identified the potential risk factors to develop sarcopenia. Diabetes, severity of COVID‐19 by means of the need of invasive ventilation, and the longer length of hospital stay were all significantly associated with an increased risk to develop sarcopenia; on the other hand, higher serum albumin levels and regular physical activity seemed to be protective factors. A good nutritional status, in particular an adequate protein intake reflected by normal albumin levels, and regular physical exercise are currently the most effective therapeutic measures to counteract sarcopenia.^27^, ^28^ The nutrients that have been most consistently linked to an improvement in sarcopenia and frailty are protein, vitamin D, essential amino acids, and their metabolites, such as β‐hydroxy β‐methylbutyrate.^28^ Current literature shows how physical exercise impacts positively on muscle physiology through systemic and local effects. From a biochemical point of view, resistance exercises increase the oxidative capacity of the muscle through the expression of some genes involved in mitochondrial function, activate satellite cells, and increase the size of type II fibres,^29^ while aerobic exercise exerts systemic effects by reducing inflammation. In fact, it is widely described in the literature that in adults enrolled in a physical exercise programme, an increase in muscle mass and function is associated with a strong reduction of inflammatory cytokines.^29^

The present work is the first large‐scale study that investigates the prevalence of sarcopenia using the new EWGSOP2 diagnostic criteria on an unselected study sample of COVID‐19 survivors. It is important to highlight that our sample includes all COVID‐19 severity degrees.^18^ It is also the first study in which sarcopenia is indicated as a possible biological substrate of the long COVID‐19 syndrome.

Limitations of the study include the lack of information on sarcopenia before acute COVID‐19 and the lack of details on sarcopenia severity. Furthermore, this is a single‐centre study with relatively large number of patients but without a control group of patients discharged for other reasons. Patients with community‐acquired pneumonia or patients with other virus disease—such as herpes or chickenpox—can also have high rate of sarcopenia and persistent symptoms, suggesting these findings could be not unique to COVID‐19. At the same time, it is really difficult to distinguish between symptoms related to long COVID‐19 and symptoms related to pre‐existing chronic diseases. However, clinical characteristics of the participants make it possible to exclude that acute illnesses were present at the time of follow‐up evaluation. Furthermore, many participants complained of myalgia and/or joint pain, and the presence of these symptoms may have potentially influenced the data. Finally, we were not able to measure a larger range of inflammatory biomolecules, and so we could not depict the full inflammatory frame in which sarcopenia might have developed in the study population. However, C‐reactive protein levels reported in the present investigation are consistent with the result obtained in a cohort of older adults with physical frailty and sarcopenia.^30^ A role for the background inflammatory milieu—remnant of SARS‐CoV‐2 infection—may also be postulated to explain the C‐reactive protein levels reported in the study. Of course, the cross‐sectional nature of the present study limits the inference of temporal/causal relationships between any inflammatory mediator and the development of sarcopenia.

Apart from these limitations, this study offers a unique opportunity to investigate the prevalence of and the risk factors for sarcopenia among an unselected population of COVID‐19 survivors adopting the new EWGSOP2 criteria. In particular, given the health implications of sarcopenia, timely detection of lower handgrip strength test may be useful in assessment of potential physical function impairment and long COVID‐19 symptoms. Cytokine storm is the key pathogenetic factor of the most severe COVID‐19 cases, which are characterized by an important catabolic component. Therefore, a greater severity of COVID‐19 will correspond to a greater risk of sarcopenia and COVID‐19 long‐term effects. Regardless of COVID‐19, the length of hospital stay is always an important risk factor for the onset of sarcopenia.^31^, ^32^ In this context, physical activity, especially if associated with adequate nutritional support, seems to be important protective factor.

In conclusion, long COVID‐19 syndrome, except for some specific cases (i.e. post‐viral pericarditis and the appearance of immune disorders triggered by viral disease), shows significant overlaps with sarcopenia syndrome. Once again, the identification of subjects with long COVID‐19 syndrome in whom sarcopenia is the cause of clinical phenotype ‘fatigue and dyspnoea’ becomes crucial because these subjects are able to earn significant benefits from interventions—nutrition and exercise—addressing muscle health. These interventions will target a function and not a pathology, revolutionizing the paradigm adopted up to now in clinical practice. An early identification of sarcopenia appears essential to prevent and to treat long COVID‐19, a real current challenge for our health system.^33^, ^34^

## Conflict of interest

None of the participants in the Gemelli Against COVID‐19 Post‐Acute Care Study Group have any conflict of interest. M.C.R. and M.R. are employees of Abbott Nutrition.

## Ethics statement

The study was approved by the Ethics Committee of the Università Cattolica del Sacro Cuore (Rome, Italy).

## Funding

This study was supported by Abbott Nutrition.
